# Supplementary material for: Molecular basis for proofreading by the unique exonuclease domain of Family-D DNA polymerases
Source: Nat Commun. 2023 Dec 14;14:8306. doi: 10.1038/s41467-023-44125-x (PMC10721889; doi:10.1038/s41467-023-44125-x)
Supplement: Supplementary file 1 — Supplementary Information [file 41467_2023_44125_MOESM1_ESM.pdf]

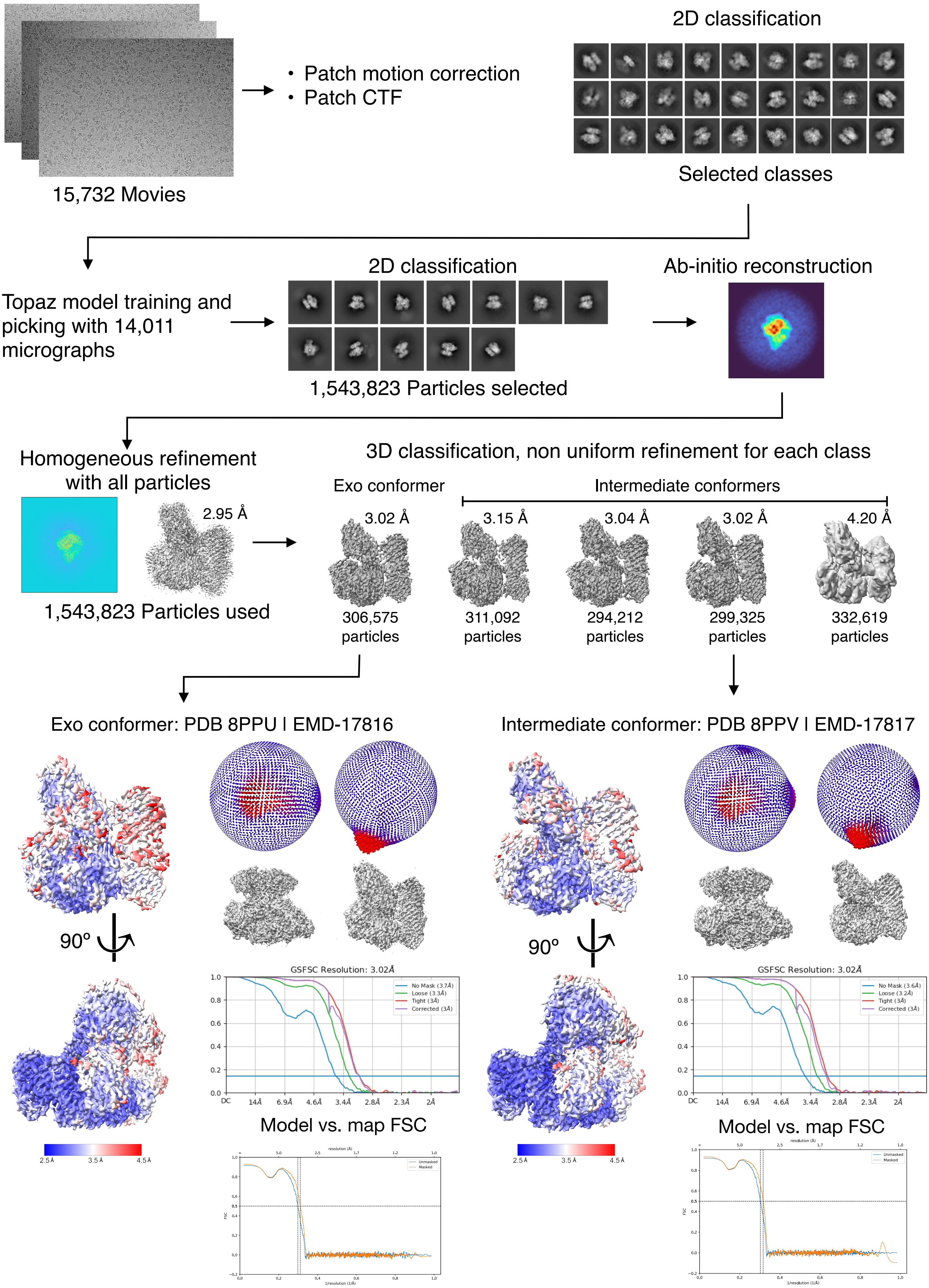

Supplementary Figure 1: Cryo-EM workflow of PolD-PCNA in complex with a DNA substrate containing three consecutive mismatches. The two selected maps were used to obtain local resolution representations and particle orientation distributions.

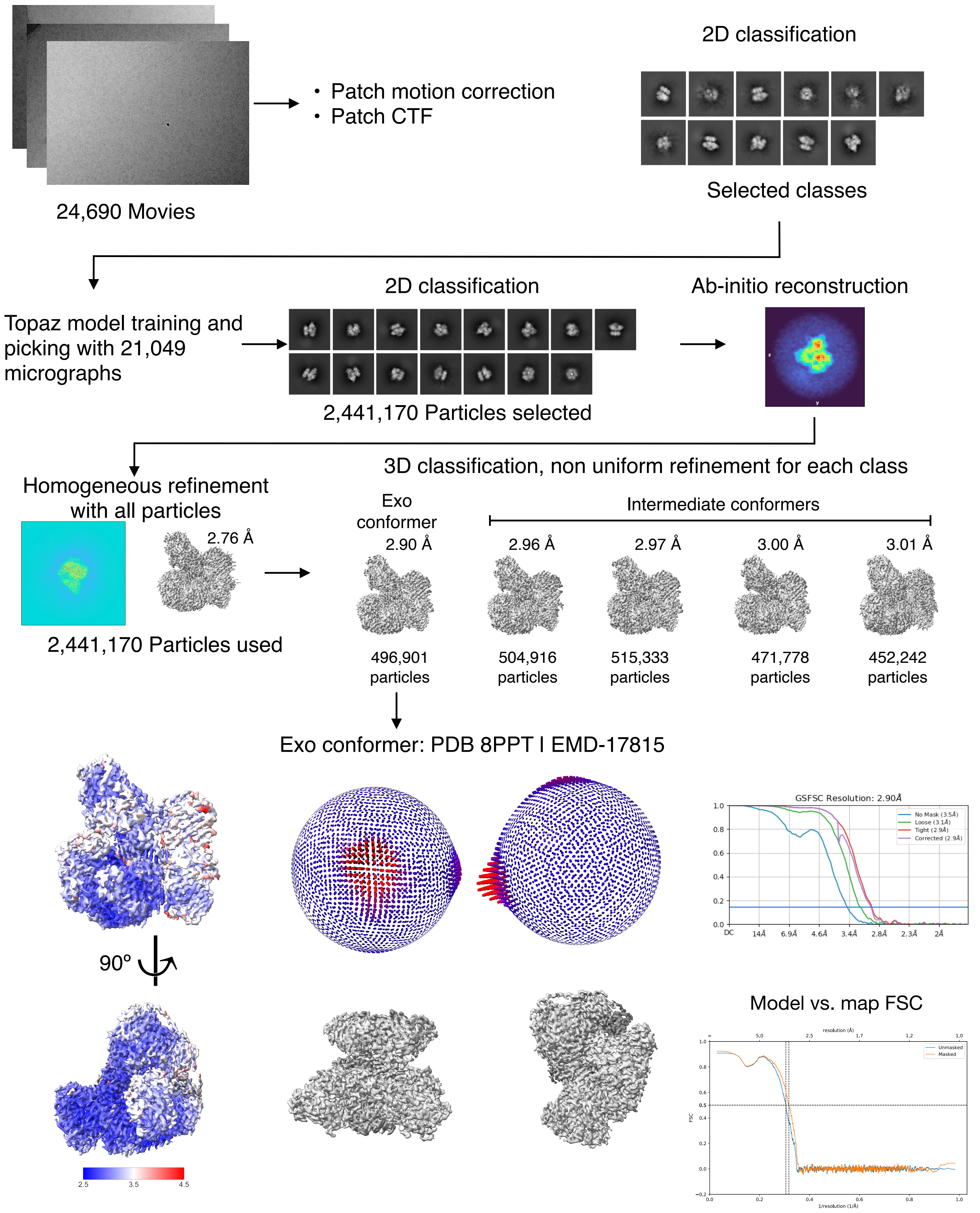

Supplementary Figure 2: Cryo-EM workflow of PolD-PCNA in complex with a DNA substrate containing a single mismatch at the second last position of the primer. The selected maps are used to obtain local resolution representations and particle orientation distributions.

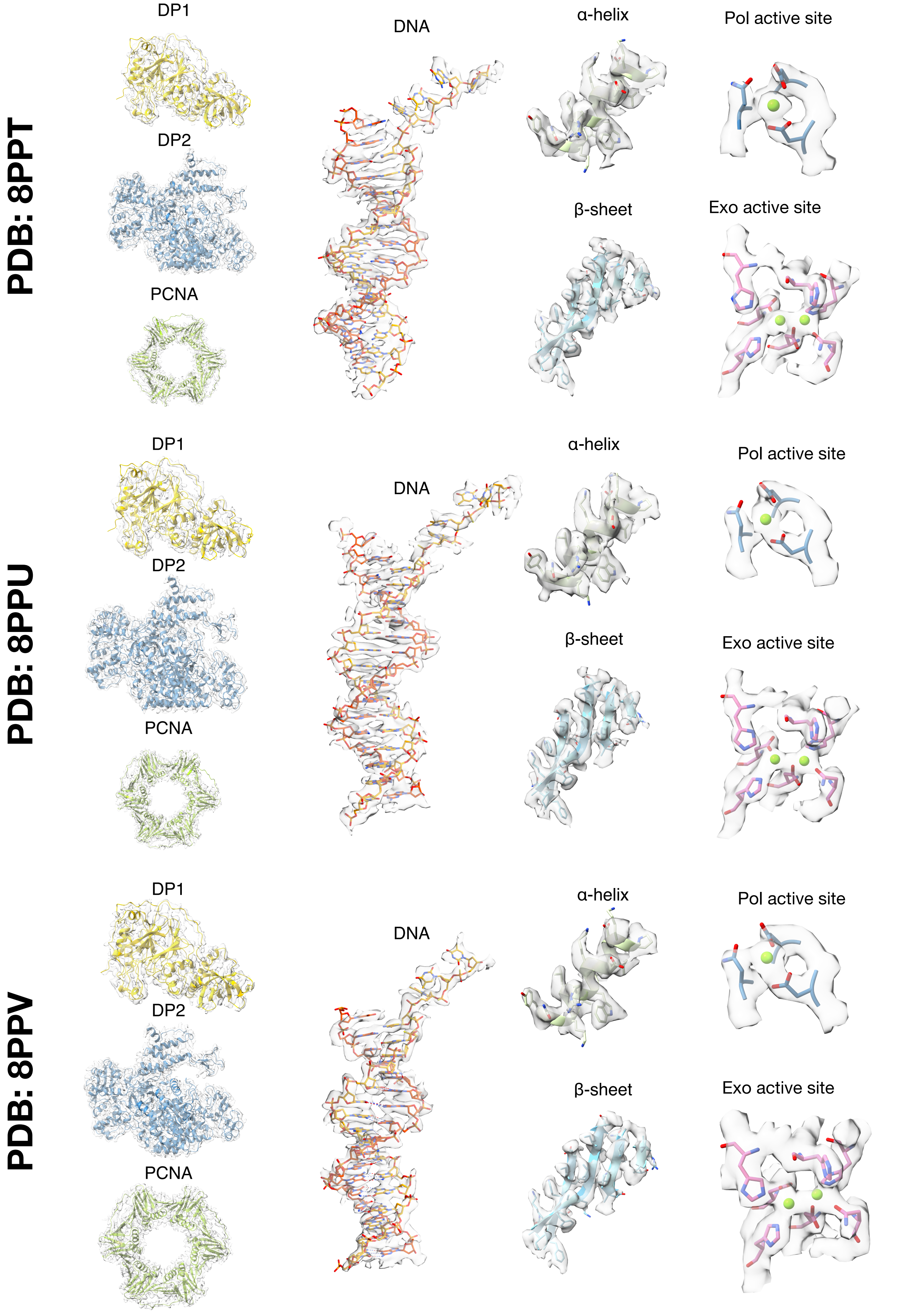

Supplementary Figure 3: Cryo-EM map at representative regions of PolD. The map contour level is 0.2 except for the active site regions, where it is 0.4.

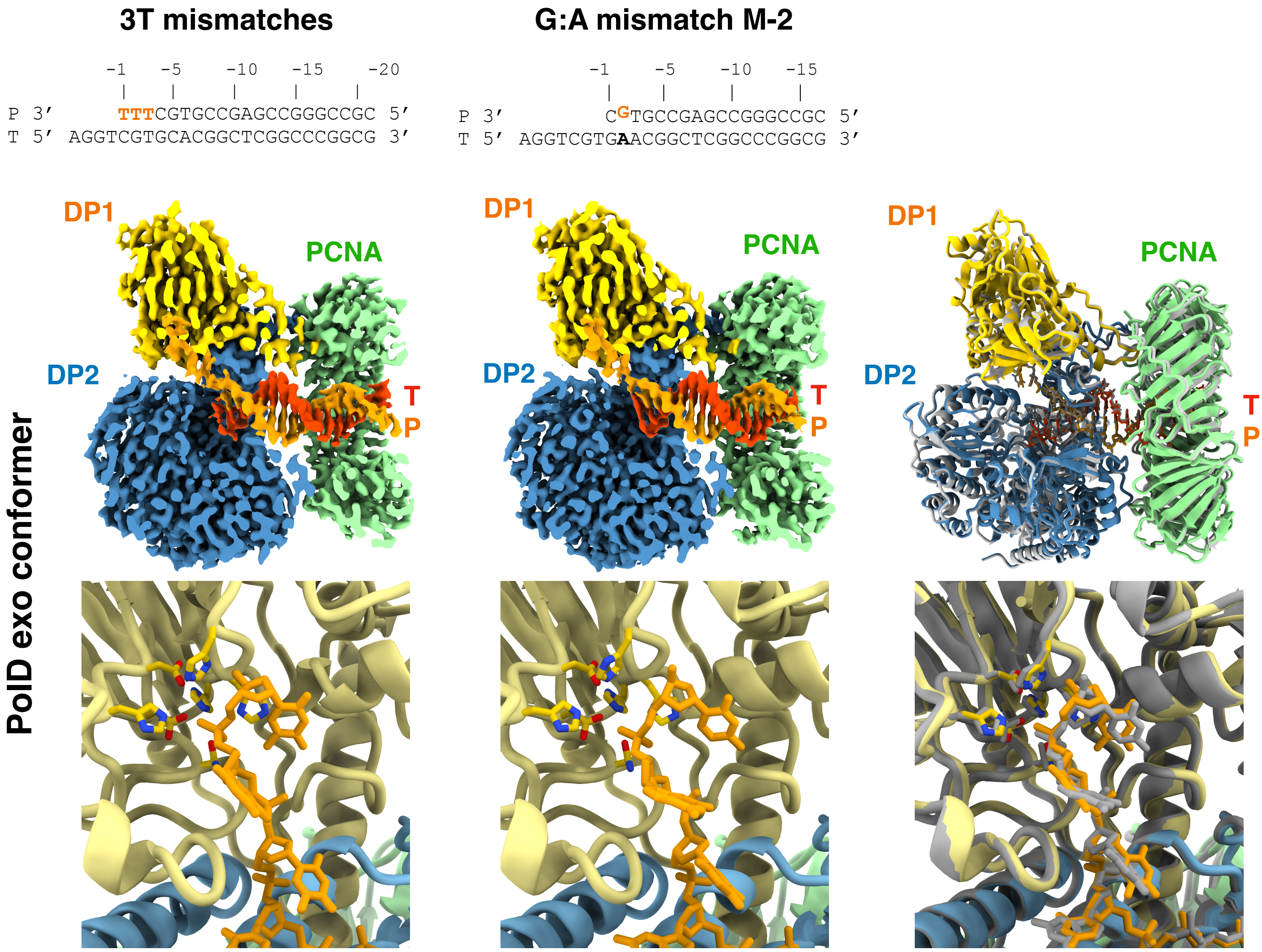

Supplementary Figure 4: Frontal cutoff view of the electron density map of PolD-PCNA in complex with the two substrates used for cryo-EM. The two substrates show the primer at the exonuclease site in the PolD Exo conformers. The superposition on the right shows illustrates the high similarity between the two structures determined with two different DNA substrates. The colored model corresponds to the one obtained with the 3T mismatches and the grey model corresponds to the MM-2 model. Root mean squared deviation: 0.253 Å across 1198 pairs of atoms

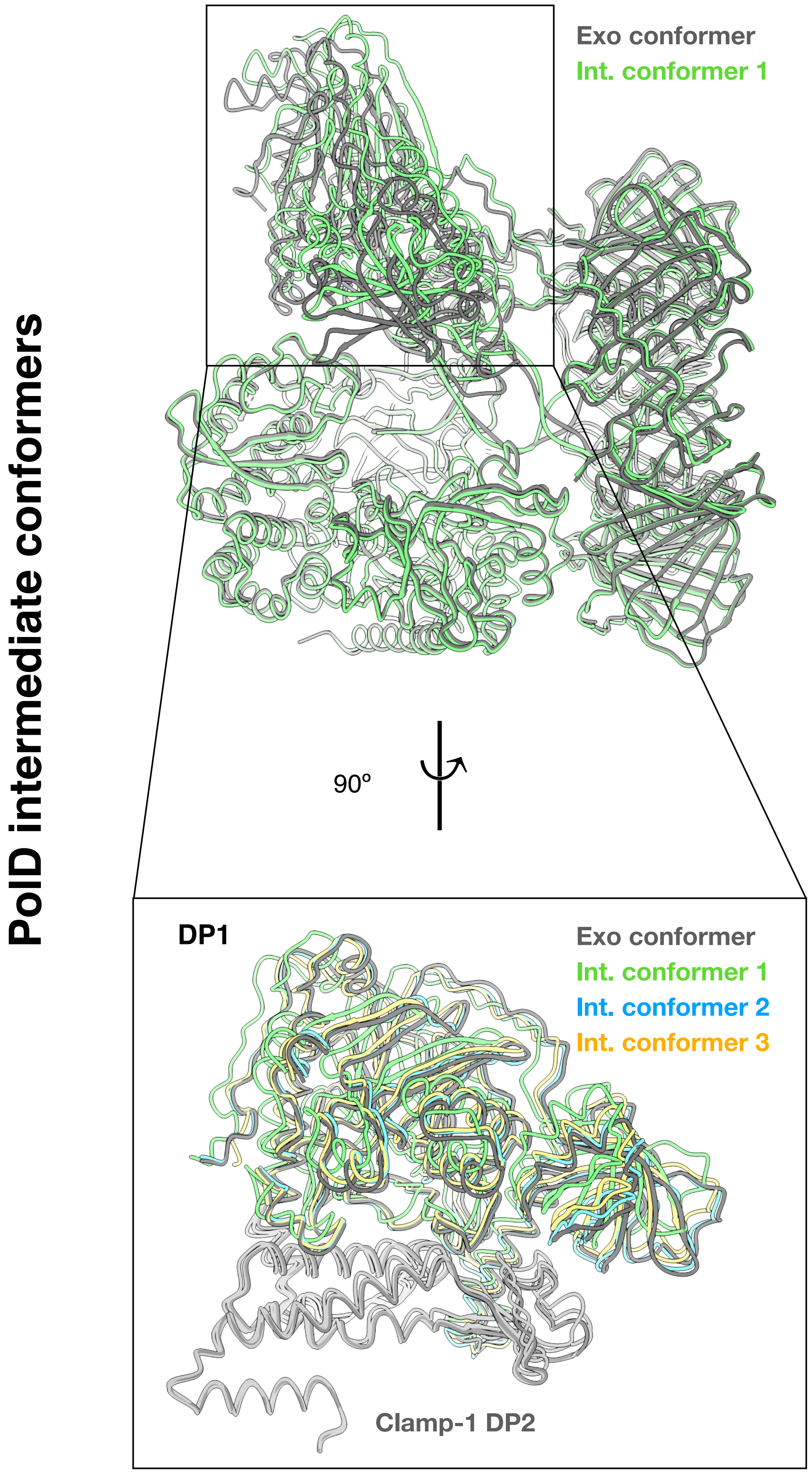

Supplementary Figure 5: (Top) Ribbon representations of the PolD Exo conformer (grey) obtained with the DNA substrate containing three consecutive mismatches (PDB: 8PPU) superimposed with the most open intermediate conformer (PDB: 8PPV) (green). (Bottom) The zoomed view shows DP1 and the DP2 clamp-1 domain from all the conformers obtained from cryo-EM 3D classification.

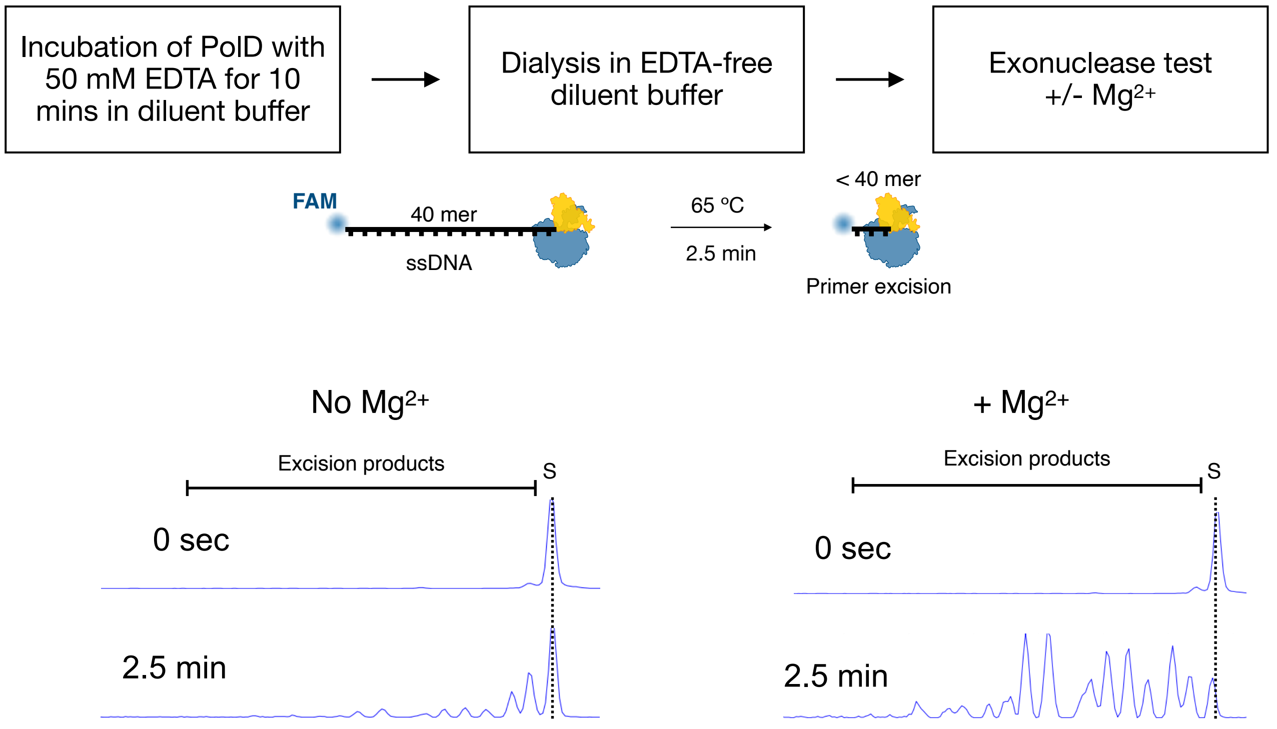

Supplementary Figure 6: Single stranded primer excision assay after incubation of PolD with EDTA followed by dialysis overnight. Treated PolD is incubated with ssDNA in the presence or absence of Mg^{2+}. The excision products are resolved by capillary electrophoresis.

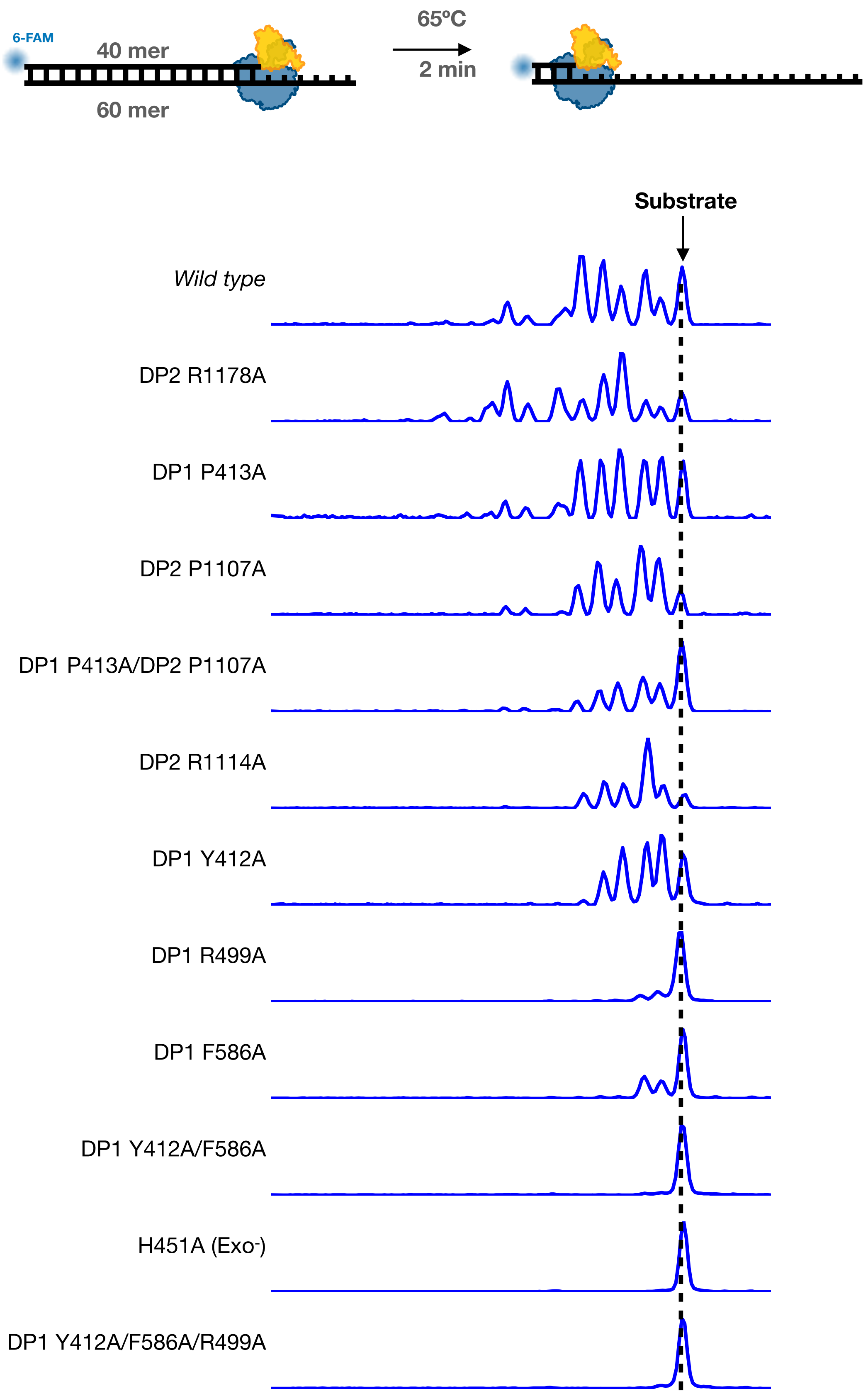

Supplementary Figure 7: Primer/ template excision with PolD 50 nM PolD (or mutant) were incubated with 20 nM primer/ template DNA in the absence of nucleotides for 2 minutes. The reaction was quenched with 50 nM EDTA and products resolved by capillary electrophoresis.

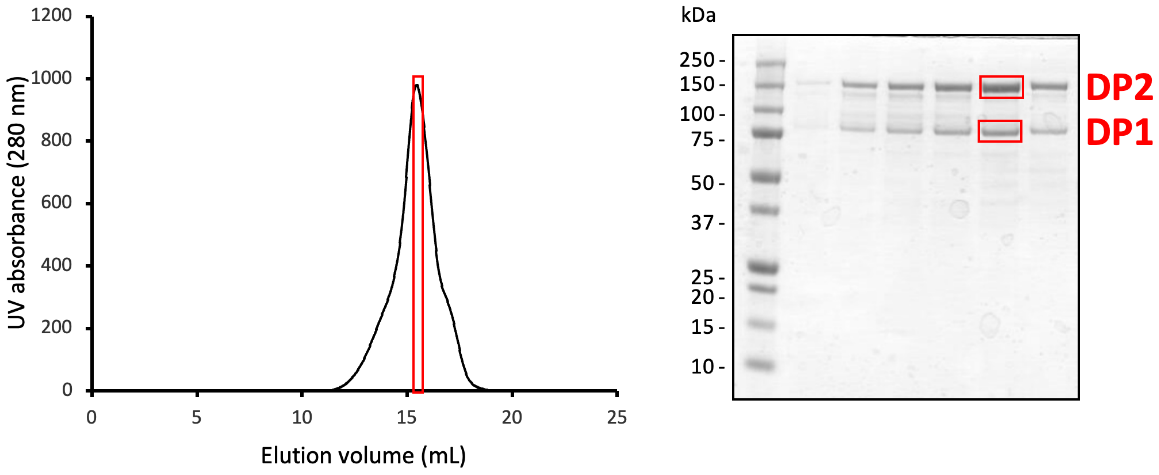

Supplementary Figure 8: Chromatogram of the size exclusion step of PolD purification for Cryo-EM, the fraction indicated in red in the chromatogram and SDA-PAGE gel was used for grid preparation.

Cryo-EM data collection, refinement, and validation statistics			
**Data collection and processing**	PolD exo conformer (TTT mismatch)	PolD Intermediate conformer (TTT mismatch)	PolD exo conformer (G:A MM-2)
**PDB entry**	8PPU	8PPV	8PPT
**EMDB entry**	EMD-17816	EMD-17817	EMD-17815
Magnification	165,000 x	165,000 x	165,000 x
Voltage (keV)	300	300	300
Electron exposure ($e^{-}/Å^{2}$)	40	40	40
Defocus range (μM)	-1 to -3	-1 to -3	-1 to -3
Pixel size (Å)	0.86	0.86	0.86
Symmetry imposed	C1	C1	C1
Initial particle images (no.)	2,627,820	2,627,820	3,487,639
Final particle images (no.)	306,575	299,325	496,901
Map resolution (Å)	3.02	3.02	2.90
FSC threshold	0.143	0.143	0.143
Map resolution range (Å)	2.5 - 4.5	2.5 - 4.5	2.5 - 4.5
**Refinement**			
Initial models used	PDB: 6T8H, AlphaFold2	PDB: 6T8H, AlphaFold2	PDB: 6T8H, AlphaFold2
Model resolution	3.3	3.3	3.2
FSC threshold	0.143	0.143	0.143
Model resolution range	2.5 - 4.5	2.5 - 4.5	2.5 - 4.5
Map sharpening B factor (Å)	105.8	108.8	105.4
**Model composition**			
Non-hydrogen atoms	19,656	19,656	19,677
Protein/DNA residues	2,376/36	2,376/36	2,376/37
Ligands	6	6	6
**B factors ($Å^{2}$)**			
Protein	62.96	70.54	66.62
Nucleotide	117.87	112.44	110.49
**R.m.s deviations**			
Bond lengths (Å)	0.012	0.003	0.009
Bond angles (°)	0.528	0.434	0.679
**Validation**			
MolProbity score	2.24	2.11	2.47
Clashscore	26.80	20.82	24.43
Poor rotamers (%)	0	0.1	2.24
**Ramachandran plot**			
Favored (%)	95.30	95.72	95.39
Allowed (%)	4.7	4.19	4.61
Disallowed (%)	0	0.08	0

Supplementary Table 1: Cryo-EM data collection, refinement, and validation statistics.

**Single stranded substrates**	**Sequence 5'-3'**	
Cryo3TP	CGCCGGGCCGAGCCGTGC**T*T*T**	
Cryo3TT	AGGTCGTGCACGGCTCGGCCCGGCG	
CryoMM-2P	CGCCGGGCCGAGCCGT**G**C	
CryoMM-2T	AGGTCGTG**A**ACGGCTCGGCCCGGCG	
Pthio-P	FAM-CCTCTAGAGTCGACCTGCAGGCCGCCGGGCAAGCAGAGA*G	
MM-P	FAM-CCTCTAGAGTCGACCTGCAGGCCGCCGGGCAAGCAGAGAG	
MM-T0	CCGACTGTGAGTAGGAAGTCCTCTCTGCTTGCCCGGCGGCCTGCAGGTCGACTCTAGAGG	
MM-T-1	CCGACTGTGAGTAGGAAGTC**A**TCTCTGCTTGCCCGGCGGCCTGCAGGTCGACTCTAGAGG	
MM-T-2	CCGACTGTGAGTAGGAAGTCC**G**CTCTGCTTGCCCGGCGGCCTGCAGGTCGACTCTAGAGG	
MM-T-3	CCGACTGTGAGTAGGAAGTCCT**A**TCTGCTTGCCCGGCGGCCTGCAGGTCGACTCTAGAGG	
MM-T-4	CCGACTGTGAGTAGGAAGTCCTC**G**CTGCTTGCCCGGCGGCCTGCAGGTCGACTCTAGAGG	
MM-T-5	CCGACTGTGAGTAGGAAGTCCTCT**A**TGCTTGCCCGGCGGCCTGCAGGTCGACTCTAGAGG	
MB-T	CCGACTGTGAGTAGGAAGTC**TTCGAA**GCTTGCCCGGCGGCCTGCAGGTCGACTCTAGAGG	
MB-P0	FAM-CCTCTAGAGTCGACCTGCAGGCCGCCGGGCAAGC**TTCGAA**	
MB-P-1	FAM-CCTCTAGAGTCGACCTGCAGGCCGCCGGGCAAGC**TTCGAG**	
MB-P-2	FAM-CCTCTAGAGTCGACCTGCAGGCCGCCGGGCAAGC**TTCGGA**	
MB-P-3	FAM-CCTCTAGAGTCGACCTGCAGGCCGCCGGGCAAGC**TTCAAA**	
MB-P-4	FAM-CCTCTAGAGTCGACCTGCAGGCCGCCGGGCAAGC**TTAGAA**	
MB-P-5	FAM-CCTCTAGAGTCGACCTGCAGGCCGCCGGGCAAGC**TGCGAA**	
MB-P-6	FAM-CCTCTAGAGTCGACCTGCAGGCCGCCGGGCAAGC**GTCGAA**	
RNA-P	FAM-CCUCUAGAGUCGACCUGCAGGCCGCCGGGCAAGCAGAGAG	
RNA-T	CCGACUGUGAGUAGGAAGUCCUCUCUGCUUGCCCGGCGGCCUGCAGGUCGACUCUAGAGG	
Kin-P	FAM-CCTCTAGAGTCGACCTGCAGGCCGCCGGGCAAGCTTCG*AA	

**Annealed substrates**	**Sequence**	Single stranded substrate
Cryo 3T	CGCCGGGCCGAGCCGTGC**TTT**<br>GCGGCCCGGCTCGGCACGTGCTGGA	Cryo3TP<br>Cryo3TT
CryoMM-2	CGCCGGGCCGAGCCGT**G**C<br>GCGGCCCGGCTCGGCA**A**GTGCTGGA	CryoMM-2P<br>CryoMM-2T
Pthio	FAM-CCTCTAGAGTCGACCTGCAGGCCGCCGGGCAAGCAGAGAG<br>GGAGATCTCAGCTGGACGTCCGGCGGCCCGTTCGTCTCTCCTGAAGGATGAGTGTCAGCC	Pthio-P<br>MM-T0
MM0	FAM-CCTCTAGAGTCGACCTGCAGGCCGCCGGGCAAGCAGAGAG<br>GGAGATCTCAGCTGGACGTCCGGCGGCCCGTTCGTCTCTCCTGAAGGATGAGTGTCAGCC	MM-P<br>MM-T0
MM-1	FAM-CCTCTAGAGTCGACCTGCAGGCCGCCGGGCAAGCAGAGA**G**<br>GGAGATCTCAGCTGGACGTCCGGCGGCCCGTTCGTCTCT**A**CTGAAGGATGAGTGTCAGCC	MM-P<br>MM-T-1
MM-2	FAM-CCTCTAGAGTCGACCTGCAGGCCGCCGGGCAAGCAGAG**A**G<br>GGAGATCTCAGCTGGACGTCCGGCGGCCCGTTCGTCTC**G**CCTGAAGGATGAGTGTCAGCC	MM-P<br>MM-T-2
MM-3	FAM-CCTCTAGAGTCGACCTGCAGGCCGCCGGGCAAGCAGA**G**AG<br>GGAGATCTCAGCTGGACGTCCGGCGGCCCGTTCGTCT**A**TCCTGAAGGATGAGTGTCAGCC	MM-P<br>MM-T-3
MM-4	FAM-CCTCTAGAGTCGACCTGCAGGCCGCCGGGCAAGCAG**A**GAG<br>GGAGATCTCAGCTGGACGTCCGGCGGCCCGTTCGTC**G**CTCCTGAAGGATGAGTGTCAGCC	MM-P<br>MM-T-4
MM-5	FAM-CCTCTAGAGTCGACCTGCAGGCCGCCGGGCAAGCA**G**AGAG<br>GGAGATCTCAGCTGGACGTCCGGCGGCCCGTTCGT**A**TCTCCTGAAGGATGAGTGTCAGCC	MM-P<br>MM-T-5
DNA-P/RNA-T	FAM-CCTCTAGAGTCGACCTGCAGGCCGCCGGGCAAGCAGAGAG<br>GGAGAUCUCAGCUGGACGUCCGGCGGCCCGUUCGUCUCUCCUGAAGGAUGAGUGUCAGCC	MM-P<br>RNA-T
RNA-P/DNA-T	FAM-CCUCUAGAGUCGACCUGCAGGCCGCCGGGCAAGCAGAGAG<br>GGAGATCTCAGCTGGACGTCCGGCGGCCCGTTCGTCTCTCCTGAAGGATGAGTGTCAGCC	RNA-P<br>MM-P
RNA-P/T	FAM-CCUCUAGAGUCGACCUGCAGGCCGCCGGGCAAGCAGAGAG<br>GGAGAUCUCAGCUGGACGUCCGGCGGCCCGUUCGUCUCUCCUGAAGGAUGAGUGUCAGCC	RNA-P<br>RNA-T
MB-0	FAM-CCTCTAGAGTCGACCTGCAGGCCGCCGGGCAAGCTTCGAA<br>GGAGATCTCAGCTGGACGTCCGGCGGCCCGTTCGAAGCTTCTGAAGGATGAGTGTCAGCC	MB-P0<br>MB-T
MB-1	FAM-CCTCTAGAGTCGACCTGCAGGCCGCCGGGCAAGCTTCGAG<br>GGAGATCTCAGCTGGACGTCCGGCGGCCCGTTCGAAGCTTCTGAAGGATGAGTGTCAGCC	MB-P-1<br>MB-T
MB-2	FAM-CCTCTAGAGTCGACCTGCAGGCCGCCGGGCAAGCTTCGGA<br>GGAGATCTCAGCTGGACGTCCGGCGGCCCGTTCGAAGCTTCTGAAGGATGAGTGTCAGCC	MB-P-2<br>MB-T
MB-3	FAM-CCTCTAGAGTCGACCTGCAGGCCGCCGGGCAAGCTTCAAA<br>GGAGATCTCAGCTGGACGTCCGGCGGCCCGTTCGAAGCTTCTGAAGGATGAGTGTCAGCC	MB-P-3<br>MB-T
MB-4	FAM-CCTCTAGAGTCGACCTGCAGGCCGCCGGGCAAGCTTAGAA<br>GGAGATCTCAGCTGGACGTCCGGCGGCCCGTTCGAAGCTTCTGAAGGATGAGTGTCAGCC	MB-P-4<br>MB-T
MB-5	FAM-CCTCTAGAGTCGACCTGCAGGCCGCCGGGCAAGCTGCGAA<br>GGAGATCTCAGCTGGACGTCCGGCGGCCCGTTCGAAGCTTCTGAAGGATGAGTGTCAGCC	MB-P-5<br>MB-T
MB-6	FAM-CCTCTAGAGTCGACCTGCAGGCCGCCGGGCAAGCGTCGAA<br>GGAGATCTCAGCTGGACGTCCGGCGGCCCGTTCGAAGCTTCTGAAGGATGAGTGTCAGCC	MB-P-6<br>MB-T
Kin-FM	FAM-CCTCTAGAGTCGACCTGCAGGCCGCCGGGCAAGCTTCGAA<br>GGAGATCTCAGCTGGACGTCCGGCGGCCCGTTCGAAGCTTCTGAAGGATGAGTGTCAGCC	Kin-P<br>MB-T
Kin-MM-1	FAM-CCTCTAGAGTCGACCTGCAGGCCGCCGGGCAAGCTTCGAA<br>GGAGATCTCAGCTGGACGTCCGGCGGCCCGTTCGAAGCTGCTGAAGGATGAGTGTCAGCC	Kin-P<br>MB-T

Supplementary Table 2: (Top) List of substrates used for cryo-EM and activity assays. (Bottom): list of annealed substrates used for cryo-EM and activity assays. Mismatches are shown in bold text.

PolD mutant	Mutation(s)		Notes
	DP1	DP2	
**pLB047**	H459A	N/A	Exo-
**pLB060**	N/A	N/A	*wild type*
**pLB079**	Y412A	N/A	
**pLB081**	Y412A / F586A	N/A	
**pLB083**	N/A	R1114A	
**pLB085**	N/A	R1178A	
**pLB087**	N/A	P1107A	
**pLB091**	R499A	N/A	
**pLB092**	P413A	N/A	
**pLB094**	P413A	P1107A	
**pLB137**	F586A	N/A	
**pLB155**	Y412A / F586A / R499A	N/A	

Supplementary Table 3: List of *P. abyssi* PolD mutants created with their respective mutations in DP1 and DP2
