# Supplementary material for: Molecular basis for proofreading by the unique exonuclease domain of Family-D DNA polymerases
Source: Nat Commun. 2023 Dec 14;14:8306. doi: 10.1038/s41467-023-44125-x (PMC10721889; doi:10.1038/s41467-023-44125-x)
Supplement: Supplementary file 3 — Description of Additional Supplementary Files [file 41467_2023_44125_MOESM3_ESM.pdf]

Description of Additional Supplementary Files

File Name: Supplementary Movie 1

Description: Molecular morphing between the PolD intermediate conformer (open, 8PPV) and the PolD exo conformer (closed, 8PPU).
