# Supplementary material for: Molecular basis for proofreading by the unique exonuclease domain of Family-D DNA polymerases
Source: Nat Commun. 2023 Dec 14;14:8306. doi: 10.1038/s41467-023-44125-x (PMC10721889; doi:10.1038/s41467-023-44125-x)
Supplement: Supplementary file 5 — Reporting Summary [file 41467_2023_44125_MOESM5_ESM.pdf]

nature portfolio

Corresponding author(s): Ludovic Sauguet, Andy Gardner, Kelly Zatopek

Last updated by author(s): Nov 27, 2023

Reporting Summary

Nature Portfolio wishes to improve the reproducibility of the work that we publish. This form provides structure for consistency and transparency in reporting. For further information on Nature Portfolio policies, see our Editorial Policies and the Editorial Policy Checklist.

Statistics

For all statistical analyses, confirm that the following items are present in the figure legend, table legend, main text, or Methods section.

n/a	Confirmed	
☐	☒	The exact sample size (*n*) for each experimental group/condition, given as a discrete number and unit of measurement
☒	☐	A statement on whether measurements were taken from distinct samples or whether the same sample was measured repeatedly
☒	☐	The statistical test(s) used AND whether they are one- or two-sided<br>*Only common tests should be described solely by name; describe more complex techniques in the Methods section.*
☒	☐	A description of all covariates tested
☒	☐	A description of any assumptions or corrections, such as tests of normality and adjustment for multiple comparisons
☐	☒	A full description of the statistical parameters including central tendency (e.g. means) or other basic estimates (e.g. regression coefficient) AND variation (e.g. standard deviation) or associated estimates of uncertainty (e.g. confidence intervals)
☒	☐	For null hypothesis testing, the test statistic (e.g. *F*, *t*, *r*) with confidence intervals, effect sizes, degrees of freedom and *P* value noted<br>*Give P values as exact values whenever suitable.*
☒	☐	For Bayesian analysis, information on the choice of priors and Markov chain Monte Carlo settings
☒	☐	For hierarchical and complex designs, identification of the appropriate level for tests and full reporting of outcomes
☒	☐	Estimates of effect sizes (e.g. Cohen's *d*, Pearson's *r*), indicating how they were calculated

Our web collection on statistics for biologists contains articles on many of the points above.

Software and code

Policy information about availability of computer code

Data collection	EPU v.3 (thermofischer) was used for Cryo-EM data collection.
Data analysis	For all structural biology data analysed in the context of this work, the following software were used: COOT v0.9.6, AlphaFold2 v2.3, Cryosparc v.4.2, Phenix v1.19-4092, APBS v1.4, DALI v5, UCSF Chimera X v1.0. All these tools are described in the literature and publicly available.

For manuscripts utilizing custom algorithms or software that are central to the research but not yet described in published literature, software must be made available to editors and reviewers. We strongly encourage code deposition in a community repository (e.g. GitHub). See the Nature Portfolio guidelines for submitting code & software for further information.

Data

Policy information about availability of data

All manuscripts must include a data availability statement. This statement should provide the following information, where applicable:

- Accession codes, unique identifiers, or web links for publicly available datasets
- A description of any restrictions on data availability
- For clinical datasets or third party data, please ensure that the statement adheres to our policy

The cryo-EM maps and all-atom models of the structures we present are available as follows. Exo conformer of PolD containing a single mismatch (PDB 8PPT https://doi.org/10.2210/pdb8PPT/pdb, https://www.ebi.ac.uk/pdbe/entry/emdb/EMD-17815), exo conformer of PolD containing three consecutive mismatches

nature portfolio | reporting summary

April 2023

(PDB 8PPU https://doi.org/10.2210/pdb8PPU/pdb, https://www.ebi.ac.uk/pdbe/entry/emdb/EMD-17816), and an intermediate conformer of PolD containing three consecutive mismatches (PDB 8PPV https://doi.org/10.2210/pdb8PPV/pdb, https://www.ebi.ac.uk/pdbe/entry/emdb/EMD-17817).

Research involving human participants, their data, or biological material

Policy information about studies with human participants or human data. See also policy information about sex, gender (identity/presentation), and sexual orientation and race, ethnicity and racism.

Reporting on sex and gender	N/A
Reporting on race, ethnicity, or other socially relevant groupings	N/A
Population characteristics	N/A
Recruitment	N/A
Ethics oversight	N/A

Note that full information on the approval of the study protocol must also be provided in the manuscript.

Field-specific reporting

Please select the one below that is the best fit for your research. If you are not sure, read the appropriate sections before making your selection.

☒ Life sciences ☐ Behavioural & social sciences ☐ Ecological, evolutionary & environmental sciences

For a reference copy of the document with all sections, see nature.com/documents/nr-reporting-summary-flat.pdf

Life sciences study design

All studies must disclose on these points even when the disclosure is negative.

Sample size	For each capillary electrophoresis experiments, about 6000 points are recorded. For time course reactions, the experiments were replicated 3 times independently. The displayed rates are the average of the three experimental replicates ± standard deviation.
Data exclusions	No data were excluded.
Replication	All kinetic course enzymatic reaction studies were replicated 3 times, and all attempts at replication were succesfull.
Randomization	For kinetic enzymatic studies, randomization of the data is not easily applicable. Randomization is not applicable for these enzymatic studies, which aim at comparing the kinetic rates of mutants that were selected to specifically target the active site. For the Cryo-EM dataset: The resolution of the cryo-EM maps was determined by splitting all particles into two random particle half sets and assessing their Fourier Shell Correlation graphs, with a cut off at the golden standard 0.143.
Blinding	Blinding, which refers to the concealment of group allocation from one or more individuals involved in a clinical research study is not easily applicable to our enzymatic experiments. Indeed, identifying the target enzyme variant and the control is important for the technical set-up of these experiments.

Reporting for specific materials, systems and methods

We require information from authors about some types of materials, experimental systems and methods used in many studies. Here, indicate whether each material, system or method listed is relevant to your study. If you are not sure if a list item applies to your research, read the appropriate section before selecting a response.

Materials & experimental systems

n/a	Involved in the study	
☒	☐	Antibodies
☒	☐	Eukaryotic cell lines
☒	☐	Palaeontology and archaeology
☒	☐	Animals and other organisms
☒	☐	Clinical data
☒	☐	Dual use research of concern
☒	☐	Plants

Methods

n/a	Involved in the study	
☒	☐	ChIP-seq
☒	☐	Flow cytometry
☒	☐	MRI-based neuroimaging
